# Classification of Parkinson’s disease and essential tremor based on balance and gait characteristics from wearable motion sensors via machine learning techniques: a data-driven approach

**DOI:** 10.1186/s12984-020-00756-5

**Published:** 2020-09-11

**Authors:** Sanghee Moon, Hyun-Je Song, Vibhash D. Sharma, Kelly E. Lyons, Rajesh Pahwa, Abiodun E. Akinwuntan, Hannes Devos

**Affiliations:** 1 Department of Physical Therapy, Ithaca College, Ithaca, NY USA; 2 Department of Physical Therapy and Rehabilitation Science, University of Kansas Medical Center, Kansas City, KS USA; 3 Department of Information Technology, Jeonbuk National University, Jeonju, South Korea; 4 Department of Neurology, University of Kansas Medical Center, Kansas City, KS USA; 5 Office of the Dean, School of Health Professions, University of Kansas Medical Center, Kansas City, KS USA

**Keywords:** Parkinson’s disease, Essential tremor, Balance, Gait, Inertial motion unit, Machine learning

## Abstract

**Background:**

Parkinson’s disease (PD) and essential tremor (ET) are movement disorders that can have similar clinical characteristics including tremor and gait difficulty. These disorders can be misdiagnosed leading to delay in appropriate treatment. The aim of the study was to determine whether balance and gait variables obtained with wearable inertial motion sensors can be utilized to differentiate between PD and ET using machine learning. Additionally, we compared classification performances of several machine learning models.

**Methods:**

This retrospective study included balance and gait variables collected during the instrumented stand and walk test from people with PD (*n* = 524) and with ET (*n* = 43). Performance of several machine learning techniques including neural networks, support vector machine, k-nearest neighbor, decision tree, random forest, and gradient boosting, were compared with a dummy model or logistic regression using F1-scores.

**Results:**

Machine learning models classified PD and ET based on balance and gait characteristics better than the dummy model (F1-score = 0.48) or logistic regression (F1-score = 0.53). The highest F1-score was 0.61 of neural network, followed by 0.59 of gradient boosting, 0.56 of random forest, 0.55 of support vector machine, 0.53 of decision tree, and 0.49 of k-nearest neighbor.

**Conclusions:**

This study demonstrated the utility of machine learning models to classify different movement disorders based on balance and gait characteristics collected from wearable sensors. Future studies using a well-balanced data set are needed to confirm the potential clinical utility of machine learning models to discern between PD and ET.

## Background

Parkinson’s disease (PD) and essential tremor (ET) are common movement disorders characterized by the presence of tremor [1]. Although ET has traditionally been considered a mono-symptomatic disorder presenting with tremor, increasing evidence suggests that ET is a complex disorder with involvement of other motor and non-motor symptoms [2]. Both PD and ET can share clinical features including motor symptoms such as bradykinesia (slow movement), gait impairment and dystonia (involuntary muscle contraction), and non-motor symptoms such as cognitive impairments, sleep disturbances, depression, and anxiety [3, 4]. Diagnosis of these disorders can be challenging for clinicians due to overlapping symptoms, and these disorders are frequently confused and misdiagnosed. A past study reported that about a third of patients with PD or dystonia were misdiagnosed with ET [5]. Since misdiagnosis can prevent or delay appropriate medical care and worsen patients’ quality of life, accurate differentiation between PD and ET is important to provide optimal care.

Clinical observation of balance and gait impairments can play a major role in classifying different conditions and monitoring the progression of PD and ET. Subtle changes in gait have even been found to occur before a clinical diagnosis of PD [6, 7], Alzheimer’s disease [8], or multiple sclerosis [9], suggesting gait as a potential biomarker for neurological disorders. Balance and gait impairments are more prominent and clinically observable in PD than in ET. However, there is growing evidence suggesting gait abnormalities in patients with ET [10]. Previous studies showed balance and gait abnormalities such as decreased cadence [11, 12], decreased gait speed [12], increased double support [11, 12], abnormalities in tandem gait [13–15], and postural instability in ET [11, 16]. These abnormalities in ET are also commonly found in PD, which contribute to misdiagnosis of the two movement disorders [5].

New technology such as video analysis, radar, sonar, and wireless sensors has emerged to assist in differential diagnosis of PD and ET [17]. For example, ultra-band wireless sensors and smartphone accelerometers have been used to detect tremor in people with PD and ET [18, 19]. These new technological advances also enable objective assessment of balance and gait through numerous devices such as body-worn inertial motion unit (IMU) sensors, 3-dimentional motion capturing systems, force plates, gait walkways, and smartphones. Many movement disorder clinics and research laboratories have started to implement these technological devices in their practices [20], particularly IMU sensors to evaluate balance and gait in PD [21, 22] . Subsequently, a vast amount of complex and non-linear data from technological devices are available for clinicians and researchers that require advanced statistical analyses.

Machine learning is widely employed to analyze large data sets produced from movement disorder clinics and research laboratories [23]; for example, to discriminate motor symptoms [24], estimate tremor severity [25], and progression of disease [26]. Among various machine learning techniques, neural network (NN) models have been utilized most due to their superior performance compared to traditional analytic methods such as logistic regression (LR) [27]. Previously, NNs have been employed in balance and gait studies to process signals from wearable devices in PD [28–31]. In addition, studies used NNs to successfully discern PD from ET using surface electromyography data [32] and assess tremor severity in PD [33]. Deep learning NN have also been used as an advanced classification method to characterize PD severity [34] and movement quality in PD [35]. Other machine learning algorithms such as support vector machine (SVM) and k-nearest neighbor (kNN) have been used to differentiate between PD and ET based on IMU sensors, but they mainly investigated upper body tremors [36–39]. To our knowledge, no study has utilized machine learning techniques to differentiate between PD and ET based on data collected from balance and gait characteristics from wearable IMU sensors.

Therefore, this data-driven study primarily aimed to examine whether balance and gait characteristics obtained from IMU sensors can distinguish between PD and ET via machine learning. Additionally, we aimed to compare and evaluate different machine learning classification performances for differential diagnosis between PD and ET.

## Methods

### Participants

This retrospective database study includes a total of 1468 people tested at the Parkinson’s Disease and Movement Disorder Clinic of the University of Kansas Medical Center between January 3, 2017 and December 11, 2018. We excluded people if they were diagnosed with both PD and ET (*n* = 29) and/or if they had a history of deep brain stimulation surgery (*n* = 468). For those who visited the clinic more than once during the study period (*n* = 628), we only included the data from their first visits. Additionally, we excluded people with no data recorded due to technical error of the measuring device (*n* = 65), leaving a total of 567 people with PD or ET in the study. Among those, 524 participants were clinically diagnosed with PD (age = 66.73 ± 9.17, disease duration = 8.20 ± 5.11 years), whereas 43 were diagnosed with ET (age = 66.98 ± 9.84, disease duration = 13.83 ± 13.79 years).

### Protocol and materials

**Fig. 1:**
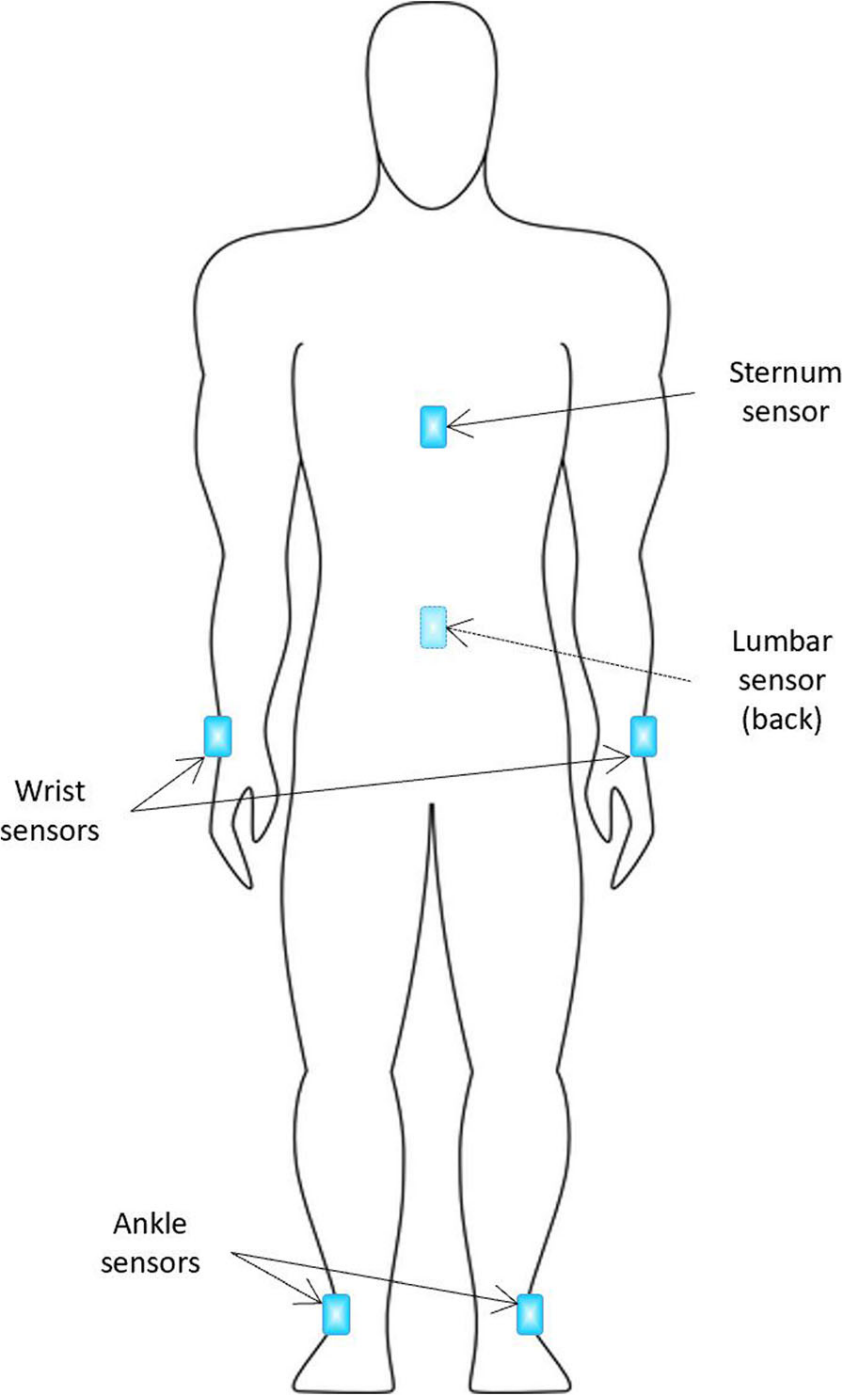
IMU sensor locations

Participants wore six IMU sensors (Opal, APDM, Inc., Portland, OR, USA) (Fig. 1). Two wrist sensors were bilaterally placed on the dorsal side of the wrist and two foot sensors were bilaterally mounted to the instep (dorsal side of metatarsus) of each foot. The sternum sensor was mounted on the sternum of the chest and the lumbar sensor was mounted to the posterior side at the level of the L5 region. All six sensors were firmly tightened to the designated locations using straps during testing.

**Fig. 2:**
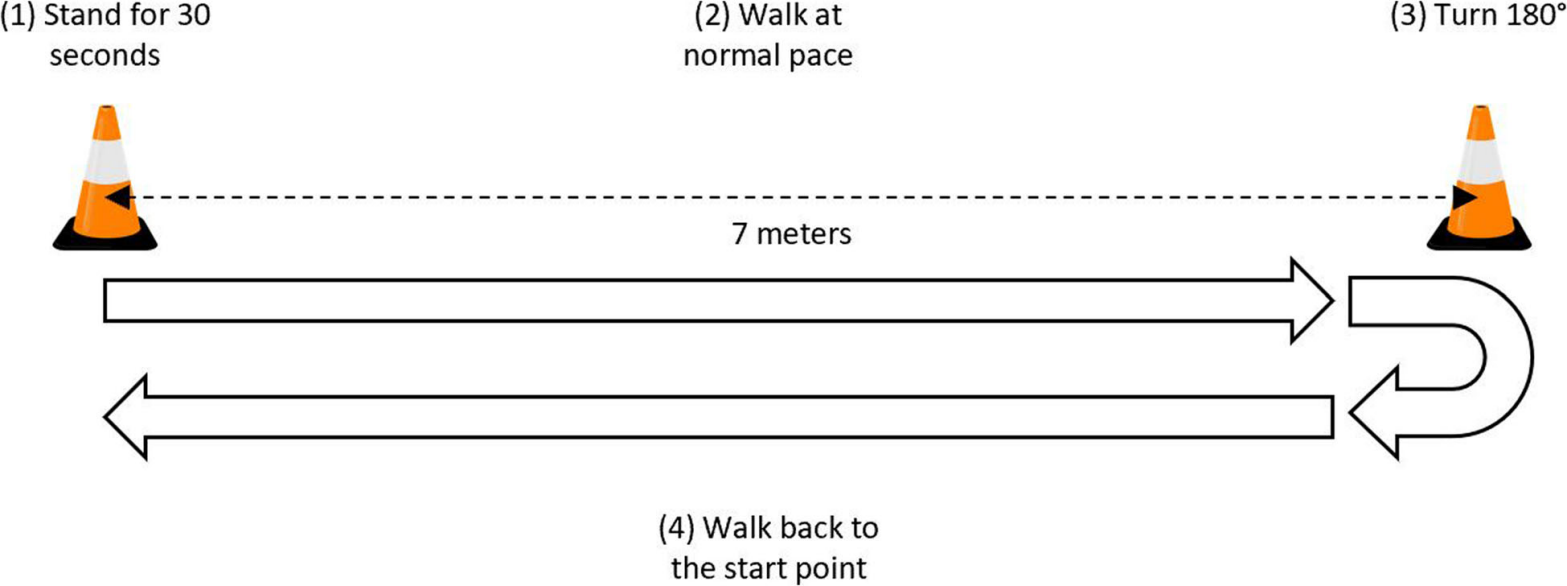
iSAW test procedure

The instrumented stand and walk (iSAW) test was administered (Fig. 2). During the iSAW test, participants were instructed to stand still for 30 s, walk straight for 7 m at a comfortable speed after hearing a beep, turn 180° around at the end of 7-m marker, then walk back to the start point. The iSAW test is a reliable and valid balance and gait measure for clinical use [40–42]. All participants wore a gait belt during the iSAW test with standby assistance from the examiner.

The IMU utilized in the study contained two accelerometers (range: ± 16 g and ± 200 g, resolution: 14 and 17.5 bits, sample rate: 128 Hz), a gyroscope (range: ± 2000°/s, resolution: 16 bits, sample rate: 128 Hz), and a magnetometer (range: ± 8 G, resolution: 12 bits, sample rate: 128 Hz). A total of 130 balance and gait features were automatically computed using the Mobility Lab software (APDM, Inc., Portland, OR, USA) [42, 43]. Pre-processed balance and gait features by the Mobility Lab software were found to be accurate compared with the 3-dimensional motion tracking system result [44].

### Data analysis

#### Pre-processing data set

A total of 130 balance and gait features were automatically computed by the Mobility Lab software. Of those, 48 features with clinical relevance were included in the study based on (1) a recent review [45] that showed clinical balance and gait parameters most distinctly representing balance and gait and (2) the clinical expertise of the authors (Table 1 and Supplementary Table 1) [46]. The detailed graphical information about balance and gait features can be found in the APDM company website (https://www.apdm.com/mobility/). The ratio between PD participants (*n* = 524, 92.5%) and ET participants (*n* = 43, 7.5%) was highly imbalanced in the data set. To mitigate the effect derived from the imbalanced data, we utilized a Synthetic Minority Over-sampling Technique (SMOTE) [47], an oversampling approach to create synthetic minority class examples. SMOTE works by synthesizing new samples using data points in the minority data set. Briefly, this statistical algorithm selects data points that are close in the feature space, then creates a new sample at a randomly selected point between two nearest neighbors in the feature space. For missing values that represented 0.0003% in the data set, we used a univariate feature imputation algorithm to predict missing values in datasets before training the classification model.

**Table 1:** Gait and balance features extracted from Mobility Lab software

**Gait – lower limb**	
Cadence [left] (steps/min)	Cadence [right] (steps/min)
Double support [left] (%GC)	Double support [right] (%GC)
Gait speed [left] (m/s)	Gait speed [right] (m/s)
Lateral step variability [left] (cm)	Lateral step variability [right] (cm)
Foot strike angle [left] (degrees)	Foot strike angle [right] (degrees)
Toe off angle [left] (degrees)	Toe off angle [right] (degrees)
Single limb support [left] (%GC)	Single limb support [right] (%GC)
Stance [left] (%GC)	Stance [right] (%GC)
Step duration [left] (s)	Step duration [right] (s)
Stride length [left] (m)	Stride length [right] (m)
Swing [left] (%GC)	Swing [right] (%GC)
Terminal double support [left] (%GC)	Terminal double support [right] (%GC)
**Gait – lumbar**	
Coronal range of motion (degrees)	
Sagittal range of motion (degrees)	
Transverse range of motion (degrees)	
**Gait – trunk**	
Coronal range of motion (degrees)	
Sagittal range of motion (degrees)	
Transverse range of motion (degrees)	
**Postural sway**	
Mean velocity (m/s)	Acc - path length (m/s^2^)
Mean velocity [coronal] (m/s)	Acc - path length [coronal] (m/s^2^)
Mean velocity [sagittal] (m/s)	Acc - path length [sagittal] (m/s^2^)
Acc - RMS sway (m/s^2^)	Acc - RMS sway (degrees)
Acc - RMS sway [coronal] (m/s^2^)	Acc - RMS sway [coronal] (degrees)
Acc - RMS Sway [sagittal] (m/s^2^)	Acc - RMS sway [sagittal] (degrees)
Sway area radius [coronal] (degrees)	Acc - range (m/s^2^)
Sway area rotation (degrees)	Acc - range [coronal] (m/s^2^)
Sway area (degrees^2^)	Acc - range [sagittal] (m/s^2^)

*Abbreviation*: *Acc* Acceleration, *GC* Gait cycle, *RMS* Root mean square

#### Classification and model selection

The classification models included NN, SVM, kNN, decision tree (DT), random forest (RF), gradient boosting classifier (GB), LR, and dummy model. The dummy model was a reference model classifier that only chooses the majority class (PD) of the data set. To find out the optimal values of the hyper-parameters of the classification models, we used a stratified 3-fold cross validation with grid search strategy. Table 2 shows the hyper-parameter search spaces of each classification model.

**Table 2:** Model hyper-parameters of the classification models

Classification models	Hyper-parameter search spaces
Neural network (NN)	hidden_layer_sizes = {100, 200, 300}, learning_rate = 0.001
Support vector machines (SVM)	C = {0.01, 0.1, 1, 5, 10, 100}, kernel = {‘linear’, ‘rbf’}, gamma = {0.01, 0.1, 1, 10}, class_weight = {None, ‘balanced’}
k-nearest neighbor (kNN)	n_neighbors = {1,3,5,7,9}, weights = {‘uniform’, ‘distance’}
Decision tree (DT)	max_depth = {5, 6, 7, 8, 9, 10, 15, 20}, class_weight = {None, ‘balanced’}
Random forest (RF)	n_estimators = {20, 50, 100, 200}, class_weight = {None, ‘balanced’, ‘balanced_subsample’}
Gradient boosting (GB)	n_estimators = {20, 50, 100, 200}
Logistic regression (LR)	C = {0.01, 0.1, 1, 5, 10, 100}, penalty = {‘l1’, ‘l2’}, class_weight = {None, ‘balanced’}

Note: Adam was used for learning rate optimization of NN [43]; gamma hyper-parameter in SVM was applied when the kernel is radial basis function ‘rbf’; class_weight was applied when the oversampling approach (SMOTE) was not used. Further details about hyper-parameters used in this study can be found: NN (https://scikit-learn.org/stable/modules/generated/sklearn.neural_network.MLPClassifier.html), SVM (https://scikit-learn.org/stable/modules/generated/sklearn.svm. LinearSVC.html#sklearn.svm.LinearSVC),kNN (https://scikit-learn.org/stable/modules/generated/sklearn.neighbors.KNeighborsClassifier.html), DT (https://scikit-learn.org/stable/modules/generated/sklearn.tree.DecisionTreeClassifier.html), RF (https://scikit-learn.org/stable/modules/generated/sklearn.ensemble.RandomForestClassifier.html), GB (https://scikit-learn.org/stable/modules/generated/sklearn.ensemble.GradientBoostingClassifier.html), and LR (https://scikit-learn.org/stable/modules/generated/sklearn.linear_model.LogisticRegression.html)

#### Performance evaluation

The classification models were evaluated with accuracy (a ratio of correct prediction to total observations), recall (a ratio of correct prediction of positive cases to all observations in actual cases), precision (a ratio of correct prediction of positive cases to all positive cases), and F1 score (a harmonic mean of precision and recall). F1 score is the most commonly used performance metric of machine learning, especially when the data set is unevenly distributed [48]. Since F1 score equally weights both false positives and false negatives, it offers less biased metric compared to accuracy [49]. Of note, all performances were micro-averaged. The accuracy, and precision and recall for the F1-score were calculated as follows (TP = true positive, TN = true negative, FP = false positive, FN = false negative):

$$ Accuracy=\frac{TP+ TN}{TP+ FN+ TN+ FP} $$

$$ Recall=\frac{TP}{TP+ FN} $$

$$ Precision=\frac{TP}{TP+ FP} $$

$$ F1\  score=\frac{2}{Recall^{-1}+{Precision}^{-1}}=2\times \frac{Recall\times Precision}{Recall+ Precision} $$

## Results

**Fig. 3:**
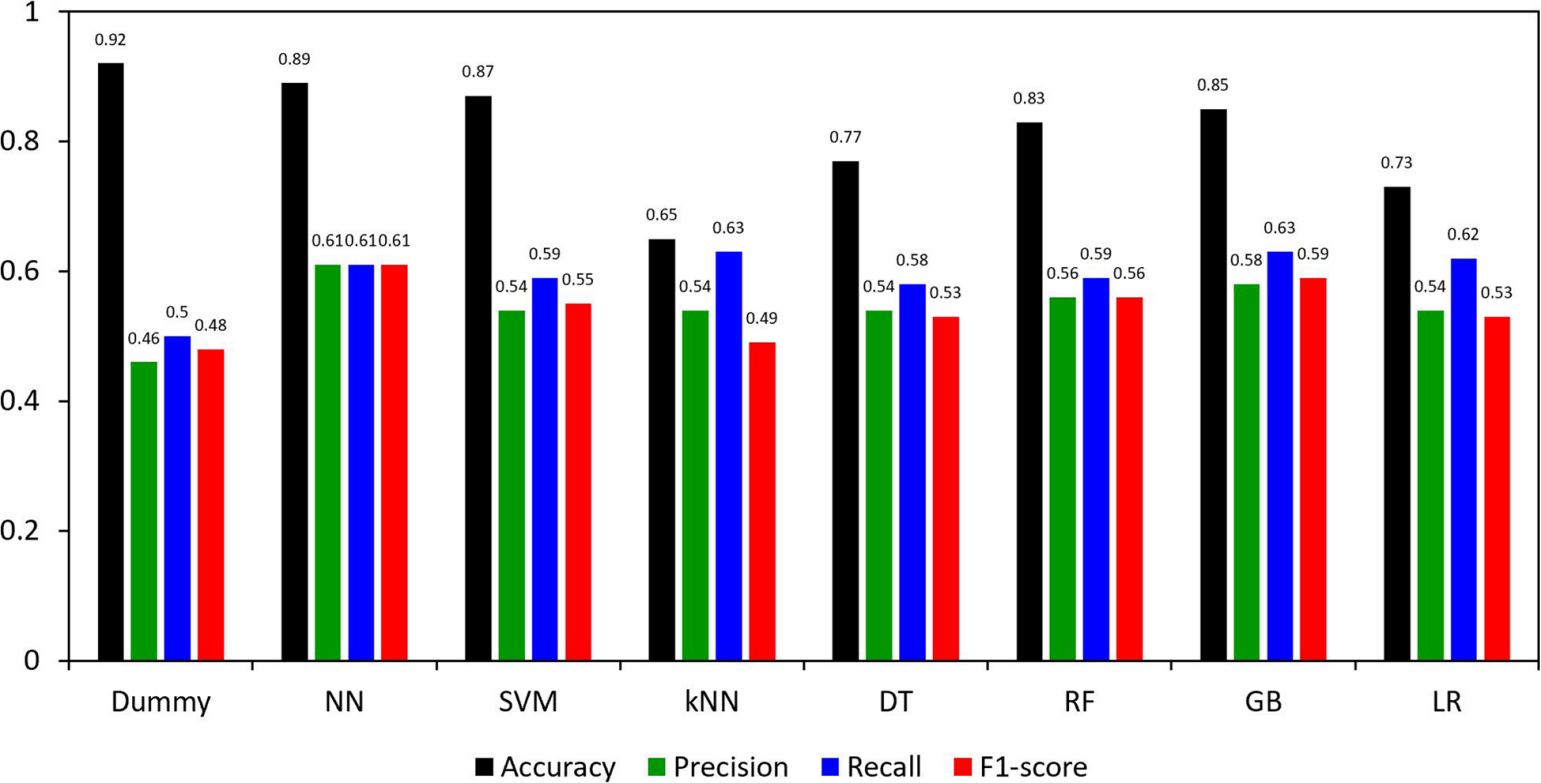
Accuracy, Precision, Recall, and F1-score of logistic regression, support vector machine, neural network, k-nearest neighbor, decision tree, random forest, and gradient boosting

The results of NN, SVM, kNN, DT, RF, GB, and LR with the oversampling approach are shown in Fig. 3. With SMOTE, (1) the accuracy of the models ranged from 0.65 (kNN) to 0.89 (NN); (2) the precision was similar across the models ranging from 0.54 (SVM, kNN, DT, and LR) to 0.61 (NN); (3) the recall ranged from 0.58 (DT) to 0.63 (kNN and GB); and (4) the F1-score ranged from 0.53 (DT and LR) to 0.61 (NN). The results without the oversampling approach can be found in Supplementary Table 2.

## Discussion

This data-driven study aimed to differentiate between two movement disorders, PD and ET based on balance and gait characteristics collected from IMU sensors using various machine learning models. Additionally, the classification performance was compared across different machine learning models.

Recent technological advances enable clinics and research laboratories to employ wearable devices in their balance and gait assessments. This allows precise measurement of balance and gait abnormalities and accurate monitoring of physical activities of daily living. However, the data produced by technological devices are often overwhelming and under-utilized due to the size and complexity of the data [50]. The current data set provides useful clinical information for balance and gait such as gait speed, cadence, and postural sway collected by wearable devices. The current results show that machine learning models can increase the utility of clinically available data collected by technological devices to classify two movement disorders. Our machine learning models outperformed (F1-scores ranging between 0.49 and 0.61) the dummy model (F1-score = 0.48) to classify the two movement disorders. Hence, our results demonstrate that machine learning models are useful to discern PD from ET using balance and gait characteristics.

In this study, with SMOTE, the NN outperformed other models in classifying PD and ET solely based on balance and gait features. The F1-score of NN was 0.61, showing the highest performance among 8 models in the analysis. The robustness of NN performance typically shows in large and complex data. Previous studies in PD have demonstrated NN as the superior machine learning technique using data collected from wearable IMU sensors in levodopa-induced dyskinesia assessment and detection [28, 29], gait abnormality classification [30], and discrimination between people with PD who underwent subthalamic stimulation and healthy controls [31]. Although the GB showed a similar performance based on the F1-score (0.59), the accuracy of GB (85%) was lower than that of the NN (89%). Accuracies of other comparison models including SVM, kNN, DT, RF, and LR ranged between 65 and 87% and their F1-scores were lower than the NN. However, particularly for this data-driven study, higher performance in accuracy does not necessarily reflect superior performance of the model, because the data set was heavily imbalanced with 92.5% of people with PD and 7.5% of people with ET. This implies that a dummy model will be 92.5% accurate in classifying PD if the model categorized each case as PD. The F1-score is a more adequate measure especially for an imbalanced data set in machine learning, because an F1-score considers both false positives and negatives and provides more weight to correctly classified samples in the minority class [51, 52].

The current study has limitations. In our study, the data set was imbalanced towards an overrepresentation of PD. To overcome this limitation, we implemented the SMOTE that generates synthetic minority class samples, which is a widely used oversampling method [53]. Our findings demonstrated that SMOTE increased the classification performance, based on F1-scores, in the majority of models in the study (Supplementary Table 2). This result may indicate the SMOTE was effective to minimize the influence of imbalanced class distribution in the current data set.

The design of the current study was a cross-sectional design including patients with clinically diagnosed PD or ET. Results of balance and gait assessment in clinic often show large variability. However, our study only focused on the outcome from one visit, which may not represent individuals’ true balance and gait performance. Thus, future research needs to combine the results from multiple visits and include a longitudinal analysis using data over time to inform the accuracy of NNs using balance and gait characteristics from wearable devices to assist in the differential diagnosis and prediction of disease progression of PD and ET.

The current study utilized balance and gait data collected from IMU sensors. Future studies combining data from IMU sensors, other sensing modalities (e.g., video, electromagnetic signal sensors), and clinical scales may allow machine learning models to provide more targeted and stratified classification (e.g., classification of PD or ET by the severity of the disease). In addition, the machine learning models derived from IMU balance and gait variables should also be employed to differentiate between different types of Parkinsonism, including progressive supranuclear palsy, multiple system atrophy, corticobasal degeneration, Lewy body dementia, or vascular Parkinsonism. One of the notable clinical features in both PD and ET is tremor [3]. Previous studies demonstrated that a smartphone accelerometer can distinguish PD and ET during upper-body tasks. Therefore, a combination of different sensor technologies that detect gait, balance, tremor, and other clinical features may offer a comprehensive and accurate diagnosis of PD and ET.

This study was performed with no control group. Future research should include a healthy control group to evaluate the accuracy of machine learning models in early diagnosis of movement disorders. The primary goal of the study was to investigate the usefulness of IMU balance and gait sensors to differentiate between PD and ET. Therefore, we did not include any participant characteristics in the machine learning models. It is likely that demographic and clinical features will improve the accuracy of the machine learning models. The current study only utilized 48 features among 130 features in the model. The size of the current data set was not big enough to include all the features as input in the machine learning models. In addition, we manually selected those 48 features based on the clinical expertise and prior-knowledge [45]. A larger sample size will allow us to include all 130 features and employ different feature selection methods in machine learning such as filter-based, wrapper-based, and embedded methods [54]. These additional selection methods may minimize the loss of features that could be influenced by human bias. The black box problem of NN models also makes it challenging to confirm which features were selected into the model [55].

Lastly, unlike past studies that utilized raw signal data captured by IMU sensors [28–31], the current study utilized pre-processed data (e.g., gait speed, sway area, cadence, etc.) from raw signal data as input variables. We opted to use the pre-processed data since these are readily available and have been tested on reliability and validity, adding to the clinical relevance of our current findings. These pre-processed balance and gait characteristics from wearable sensors have been used to evaluate movement disorder progression, fall risks, treatment efficacy, and differences between people with movement disorders and healthy controls [21]. However, we acknowledge that using raw data adds more information to the model since the pre-processing procedure might result in a significant loss of raw signal features directly from IMU sensors. In general, the performance of NN can be more precise and accurate when the model is fed more data. Thus, further examination using raw data collected from wearable IMU sensors can offer new insights that extend our current findings.

## Conclusions

Wearable sensors for balance and gait assessments can be implemented in movement disorders clinics to produce a vast amount of potentially informative data for assisting in diagnosis and monitoring disease progression. The current study showed that NN with SMOTE outperformed machine learning models and traditional logistic regression in classifying PD and ET based on the pre-processed balance and gait IMU data set. With further validation, a data-driven approach using machine learning techniques may provide a more efficient diagnostic and prognostic tool that can assist in the clinicians’ decision-making process.

## Supplementary information

**Additional file 1: Supplementary Table 1.** Balance and gait features and description.

**Additional file 2: Supplementary Table 2.** Performance across different machine learning models with and without SMOTE.

## Data Availability

The datasets analyzed during the current study are not publicly available.

## Abbreviations

DT: Decision tree
ET: Essential tremor
GB: Gradient boosting
IMU: Inertial motion unit
iSAW: Instrumented stand and walk
kNN: K-nearest neighbor
LR: Logistic regression
NN: Neural network
PCA: Principal component analysis
PD: Parkinson’s disease
RF: Random forest
SMOTE: Synthetic minority over-sampling technique
SVM: Support vector machine
