# Supplementary material for: Classification of Parkinson’s disease and essential tremor based on balance and gait characteristics from wearable motion sensors via machine learning techniques: a data-driven approach
Source: J Neuroeng Rehabil. 2020 Sep 11;17:125. doi: 10.1186/s12984-020-00756-5 (PMC7488406; doi:10.1186/s12984-020-00756-5)
Supplement: Supplementary file 1 — Additional file 1: Supplementary Table 1. Balance and gait features and description. [file 12984_2020_756_MOESM1_ESM.docx]

**Supplementary Table 1.** Gait and balance features and description [1]

| **Feature** | **Description** |
| --- | --- |
| Cadence | The number of steps per a minute period |
| Double support | The period of percentage when both feet are in contact with the ground during the gait cycle |
| Gait speed | The forward distance, 2 step lengths, travelled during the gait cycle divided by the gait cycle duration |
| Lateral step variability | In three consecutive steps, the perpendicular deviation of the middle foot placement from the line connecting the first and the third step |
| Foot strike angle | The degree when the foot initially strikes the ground |
| Toe off angle | The degree when the plantar flexion of the foot just as it leaves the ground at push off |
| Single limb support | The period of percentage when a single foot is in contact with the ground during the gait cycle |
| Stance | The percentage when the foot is on the ground during the gait cycle |
| Step duration | The duration of a step in a second |
| Stride length | The forward distance moved by a foot during the gait cycle |
| Swing | The percentage when the foot is not on the ground during the gait cycle |
| Terminal double support | The percentage of the second double support during the gait cycle |
| Lumbar - coronal range of motion | The angular range of the lumbar spine in the coronal plane (roll) |
| Lumbar - sagittal range of motion | The angular range of the lumbar spine in the sagittal plane (pitch) |
| Lumbar - transverse range of motion | The angular range of the lumbar spine in the transverse plane (yaw) |
| Trunk - coronal range of motion | The angular range of the thoracic spine in the coronal plane (roll) |
| Trunk - sagittal range of motion | The angular range of the thoracic spine in the sagittal plane (pitch) |
| Trunk - transverse range of motion | The angular range of the thoracic spine in the transverse plane (yaw) |
| Sway - Mean velocity | The mean velocity of displacement in postural sway in both the coronal and sagittal planes |
| Sway - Mean velocity [coronal] | The mean velocity of displacement in postural sway in the coronal plane |
| Sway - Mean velocity [sagittal] | The mean velocity of displacement in postural sway in the sagittal plane |
| Acceleration - path length | The acceleration of the length of the sway path in both the coronal and sagittal planes |
| Acceleration - path length [coronal] | The acceleration of the length of the sway path in the coronal plane |
| Acceleration - path length [sagittal] | The acceleration of the length of the sway path in the sagittal plane |
| Acceleration - RMS sway | The extent of postural sway calculated as the root mean square of the sway angle in both the coronal and sagittal planes |
| Acceleration - RMS sway [coronal] | The extent of postural sway calculated as the root mean square of the sway angle in the coronal plane |
| Acceleration - RMS Sway [sagittal] | The extent of postural sway calculated as the root mean square of the sway angle in the sagittal plane |
| Acceleration - range | The acceleration of postural sway in both the coronal and sagittal ranges of motion |
| Acceleration - range [coronal] | The acceleration of postural sway in the coronal range of motion |
| Acceleration - range [sagittal] | The acceleration of postural sway in the sagittal range of motion |
| Sway area radius [coronal] | The angular range of postural sway in the coronal plane |
| Sway area rotation | The angular range of postural sway in the transverse plane |
| Sway area | The area of the postural sway angle |

**Reference**

1. APDM, Inc., “Whitepaper for Mobility Lab by APDM”, APDM, Portland, Oregon, USA, 2015. Accessed on: May, 5, 2020. [Online]. Available: https://www.apdm.com/wp-content/uploads/2015/10/Whitepaper1.pdf
