# Supplementary material for: Classification of Parkinson’s disease and essential tremor based on balance and gait characteristics from wearable motion sensors via machine learning techniques: a data-driven approach
Source: J Neuroeng Rehabil. 2020 Sep 11;17:125. doi: 10.1186/s12984-020-00756-5 (PMC7488406; doi:10.1186/s12984-020-00756-5)
Supplement: Supplementary file 2 — Additional file 2: Supplementary Table 2. Performance across different machine learning models with and without SMOTE. [file 12984_2020_756_MOESM2_ESM.docx]

**Supplementary Table 2.** Performance across different machine learning models with and without SMOTE.

|  |  | **Dummy** | **NN** | **SVM** | **kNN** | **DT** | **RF** | **GB** | **LR** |
| --- | --- | --- | --- | --- | --- | --- | --- | --- | --- |
| With SMOTE | Accuracy | 0.92 | 0.89 | 0.87 | 0.65 | 0.77 | 0.83 | 0.85 | 0.73 |
|  | Precision | 0.46 | 0.61 | 0.54 | 0.54 | 0.54 | 0.56 | 0.58 | 0.54 |
|  | Recall | 0.50 | 0.61 | 0.59 | 0.63 | 0.58 | 0.59 | 0.63 | 0.62 |
|  | F1-score | 0.48 | 0.61 | 0.55 | 0.49 | 0.53 | 0.56 | 0.59 | 0.53 |
| Without SMOTE | Accuracy | 0.92 | 0.92 | 0.76 | 0.88 | 0.77 | 0.90 | 0.92 | 0.68 |
|  | Precision | 0.46 | 0.68 | 0.55 | 0.68 | 0.56 | 0.55 | 0.63 | 0.54 |
|  | Recall | 0.50 | 0.56 | 0.62 | 0.54 | 0.61 | 0.53 | 0.51 | 0.61 |
|  | F1-score | 0.48 | 0.58 | 0.54 | 0.54 | 0.55 | 0.53 | 0.50 | 0.50 |

Abbreviations: Dummy = reference classifier (when the model chooses only PD), LR = logistic regression, SVM = support vector machine, NN = neural network, kNN = k-nearest neighbor, RF = random forest, GB = gradient boosting, SMOTE = synthetic minority over-sampling technique.
